# Relative Energy Deficiency in Sport—Multidisciplinary Treatment in Clinical Practice

**DOI:** 10.3390/nu17020228

**Published:** 2025-01-09

**Authors:** Andrea Meyer, Daniel Haigis, Bea Klos, Stephan Zipfel, Gaby Resmark, Katharina Rall, Katharina Dreser, Daniela Hagmann, Andreas Nieß, Christine Kopp, Isabelle Mack

**Affiliations:** 1 Department of Psychosomatic Medicine and Psychotherapy, University Hospital Tübingen, 72076 Tübingen, Germany; andrea.meyer@med.uni-tuebingen.de (A.M.); bea.klos@med.uni-tuebingen.de (B.K.); stephan.zipfel@med.uni-tuebingen.de (S.Z.); gaby.resmark@med.uni-tuebingen.de (G.R.); isabelle.mack@uni-tuebingen.de (I.M.); 2 Department of Sports Medicine, University Hospital Tübingen, 72076 Tübingen, Germany; daniel.haigis@med.uni-tuebingen.de (D.H.); andreas.niess@med.uni-tuebingen.de (A.N.); 3 Department of Womens’ Health, University of Tübingen, 72076 Tübingen, Germany; katharina.rall@med.uni-tuebingen.de (K.R.); katharina.dreser@med.uni-tuebingen.de (K.D.); 4 Department of Child and Adolescent Psychiatry, Psychosomatics and Psychotherapy, University Hospital Tübingen, 72076 Tübingen, Germany; daniela.hagmann@med.uni-tuebingen.de; 5 German Center for Mental Health, 72076 Tübingen, Germany

**Keywords:** relative energy deficiency in sport, athletes, treatment, eating disorder, diet

## Abstract

Background/Objectives: The primary aim of this study was to characterize athletes approaching an outpatient interdisciplinary and multidisciplinary consultation structure for athletes with a suspected relative energy deficiency in sport (REDs) cross-sectionally and longitudinally to prove treatment efficacy. Methods: Data of 58 athletes suspected of REDs were collected at the onset (t_0_) and completion (t_1_) of interdisciplinary and multidisciplinary REDs treatment (clinical practice) between January 2019 and December 2022. The data included extracted information from medical records, anthropometric characteristics, physical performance diagnostics, laboratory values, dietary records, and partially gynecological and psychosomatic diagnostics. Results: The sample primarily consisted of female athletes (97%) under 18 years of age (66%) who were underweight with a body mass index (BMI) < 18.5 kg/m^2^ and BMI percentile below the 10th percentile for their age and gender-specific norms (59%), and experienced menstrual disorders (93%). The dietary behavior is characterized by plant-based and low-energy-dense foods. Eating disorders (anorexia nervosa and bulimia nervosa) were diagnosed in 40% of the athletes according to the International Statistical Classification of Diseases and Related Health Problems 11th revision criteria. During the program, 64% of the athletes exhibited a mean weight gain of 7 (±6) kg (*p* < 0.001), excluding those still undergoing treatment (36%). Conclusions: The proposed interdisciplinary and multidisciplinary treatment approach proved effective and holds promise for future evidence-based developments in REDs treatment.

## 1. Introduction

The International Olympic Committee prioritizes the physical health and well-being of athletes. However, male and female athletes in various sports may be at risk for developing the Relative Energy Deficiency in Sport (REDs) [1,2]. The Relative Energy Deficiency in Sport is caused by the exposure to problematic and adaptable low energy availability (LEA), resulting in negative effects on an athlete’s health and performance implications [2]. The LEA is induced by intentional or unintentional changes in energy intake and energy expended through exercise, favoring the latter, and has serious athletic performance and health consequences [1,2]. Potential REDs health and performance outcomes, resulting from problematic LEA, range from impaired reproductive function (e.g., amenorrhea among females and reduced testosterone among males), impaired bone health (e.g., bone stress injuries), impaired hematological status to reduced skeletal muscle function, diminished performance, and decreased training response [2,3].

Amenorrhea is common among active female athletes, affecting over 50% of endurance runners and athletes in aesthetic-focused sports, like rhythmic gymnastics [4]. Reasons for athletes’ inadequate energy availability can be a lack of nutritional skills, deliberate weight management for athletic performance or aesthetics [5], or eating disorders like anorexia nervosa (AN) and bulimia nervosa (BN). Eating disorders are more prevalent in athletes than in non-athletes and can negatively influence an athlete’s health and performance [6,7]. The prevalence of eating disorders varies significantly between different types of sports [8,9]. Particularly, sports disciplines involving endurance (e.g., long-distance running, triathlon, and cycling) and aesthetics (e.g., dance, figure skating, and artistic gymnastics) as well as sports where a low body weight or leanness provides a competitive advantage, can contribute to the development of eating disorders [9,10]. Enhanced disordered eating behaviors are an increasing challenge in managing REDs, as they may be an important risk factor for developing REDs [2]. The LEA and REDs predominantly affect physically active females [11]. Especially female and young athletes tend to be more concerned about their body weight and are at a higher risk of engaging in restrictive dieting, experiencing body dissatisfaction, and developing eating disorders [5,11]. To diagnose REDs, the International Olympic Committee has developed a screening tool, the “Clinical Assessment Tool Version 1 (CAT1)”, to assess the risk of existing REDs [12], and its updated version, CAT2 [13]. The three-stage CAT2 model is intended for the early identification of athletes at risk and/or with REDs to initiate quick and adequate treatment, depending on the severity/risk of the athlete. The risk assessment of CAT2 involves a four-level traffic light system (green, yellow, orange, and red), using primary and secondary indicators to assess the eligibility for participation in training and competition. Athletes in the green category can continue sports without treatment, whereby athletes in the yellow and orange category need periodic monitoring with partial training adjustments. Athletes in the red category require immediate treatment, mostly accompanied by abstinence from sports. The primary goal is to restore the energy availability in REDs athletes [2,13]. As yet, information on the practical use of both CATs is limited. Complete agreement on REDs risk assessment was 62% CAT2 at eight independently expert opinions in five case studies [14].

The treatment of REDs can only be achieved in an interdisciplinary and comprehensive team approach to address the athlete’s individual and heterogeneous needs [2,13], but standardized therapy procedures were not available yet [12]. Most research in this area continued to be conducted in adult athletes [2]. Knowledge on the organization and the procedures of outpatient interdisciplinary consultation structures for athletes is limited and the population of athletes seeking help with suspected REDs has not been well described [1,2,15].

The primary aim of this study was to characterize athletes approaching an outpatient interdisciplinary and multidisciplinary consultation structure for athletes with suspected REDs cross-sectionally and longitudinally to prove treatment efficacy.

## 2. Materials and Methods

The study was approved by the Ethics Committee of the medical faculty for the University Tübingen, Germany (080/2022BO2).

### 2.1. Study Information and Participants

The University Hospital Tübingen offers an interdisciplinary and multidisciplinary outpatient consultation program for athletes with suspected REDs. Athletes contact the Department of Sports Medicine where the first anamnesis and diagnostics are performed. Further interdisciplinary diagnostics and treatments are carried out by the Dept. of Gynecology, Psychosomatic Medicine and Child and Youth Psychiatry, and Dietary Counseling. Athletes with suspected REDs were eligible if they were presented between January 2019 and December 2022 and were suspicious for REDs, due to symptomatic abnormalities, based on the available data. These athletes had symptoms, such as being underweight (defined as a body mass index (BMI) < 18.5 kg/m^2^ for athletes over 18 years and a BMI below the 10th percentile for their age and gender specific norms for athletes under 18 years [16]), low body fat (defined as body fat < 5% for men and body fat < 12% for women according to the suggested critical values of the consensus statement [17]), weight loss in the last six months, menstrual disorders based on self-reports and/or diagnostics at the Dept. of Gynecology (e.g., primary and secondary amenorrhea), eating disorders (diagnosis according to the International Statistical Classification of Diseases and Health Problems Version 11 (ICD-11) criteria), frequent infections, and bone health diseases (e.g., osteoporosis, (stress) fractures or bone edema), as described in the REDs consensus statement [2,18]. Characteristics of eating disorders are defined in the ICD-11 by the World Health Organization [19]. The ICD-11 diagnostic criteria for AN are as follows: a low body weight (a BMI < 18.5 kg/m^2^ in adults, and a BMI percentile under the 5th percentile in children and adolescents) and persistent pattern of behaviors to prevent the restoration of a normal weight (restrictive eating, purging behaviors, and excessive exercise) [19].

### 2.2. Study Procedure

**Figure 1:**
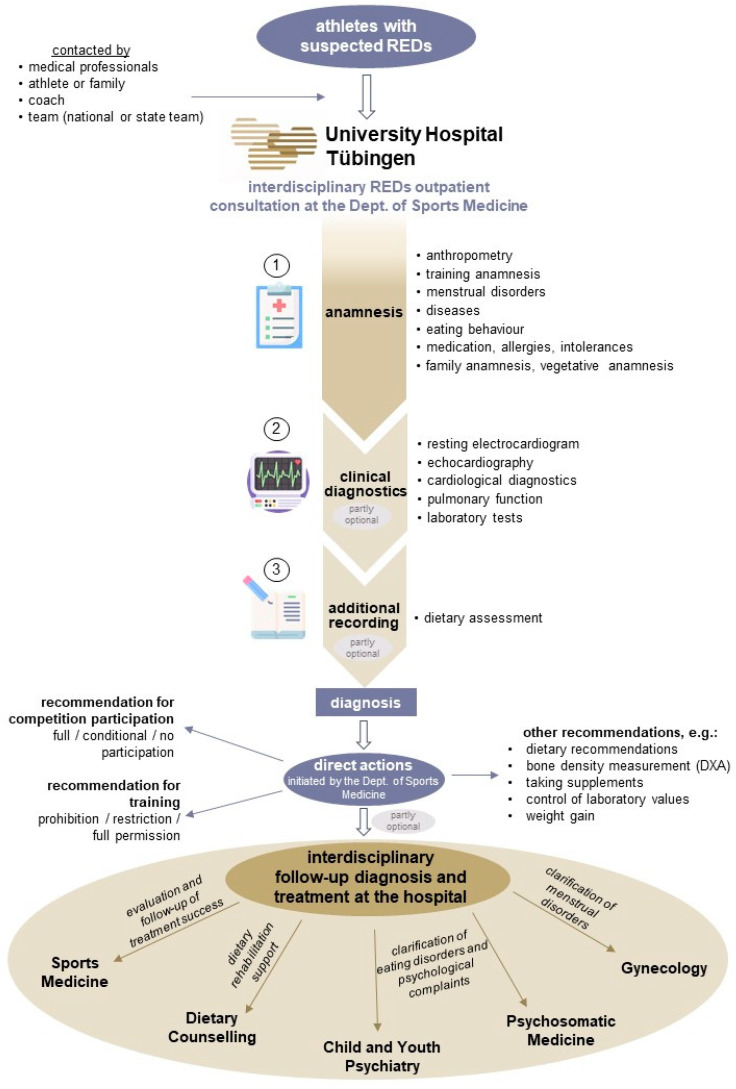
Graphical representation of the procedure of the interdisciplinary REDs outpatient consultation at the Dept. of Sports Medicine at the University Hospital Tübingen. (1) *Anamnesis*: anthropometry (height, weight, and skinfold measurements); training history (training hours and type of sport); menstrual disorders (primary and secondary amenorrhea); diseases: (fractures, bone stress injuries, (pre-)diseases, and bone edema); and eating behavior (diet form). (2) *Clinical diagnostics*: cardiological diagnostics (ergometry); pulmonary function (spirometry). Abbreviations: Dept.: department, DXA: dual-energy X-ray absorptiometry, REDs: relative energy deficiency in sport.

In 2019, a multidisciplinary outpatient consultation with clear procedures was established at the University Hospital Tübingen for athletes with REDs symptoms (Figure 1).

### 2.3. Data Collection

The following data were systematically extracted and analyzed from the athlete’s medical records using a structured procedure: demographic information (age and sex), anthropometric measurements (weight, height, body mass index or BMI percentiles, and z-scores [16]), and body composition parameters. Body composition was assessed using 3-site skinfold thickness measurements (triceps, subscapular, and paraumbilical) obtained via calipers, and body fat percentages were calculated based on Lohman’s equation as described by Lohman (1981) and Durnin et al. (1974) [20,21]. Additional anthropometric data included the waist circumference. Sports-related background information was documented, including the team membership, type of sport, and average training hours per week. Vegetative anamnesis was collected through direct interviews, covering topics such as diagnosed eating disorders (according to ICD-11 criteria), relative energy deficiency in sport (REDs), menstruation history, dietary habits, and pre-existing diseases. Blood parameters were analyzed as detailed in Supplementary Material S1. Recommendations for training, competition participation, interdisciplinary and follow-up treatment, and the frequency of appointments at different departments were documented. Dietary protocols were analyzed if available to assess the frequency of meals and food groups, and the evaluation of food in terms of the energy density (Supplementary Material S1). To investigate the effectiveness of the treatment, characteristics of the athletes who had completed their treatment before the year 2023 were compared between t_0_ (before treatment) and t_1_ (end of treatment), regardless of the treatment duration.

Based on the extracted data, the REDs risk was calculated for CAT1 (active during data assessment period [12]). In short, CAT1 was a three-level traffic light system categorizing the REDs risk into high risk (red light), moderate risk (yellow light), and no risk (green light) as described by the International Olympic Committee [12]. Athletes in the red-light risk category should not participate in sports and competition, athletes categorized in the yellow-light risk category may compete once medically cleared under supervision, while those in the green-light risk category are recommended for full sport participation [2,18]. High-risk criteria include AN or other serious eating disorders, other serious medical conditions related to LEA, or extreme weight loss techniques. Moderate risk criteria involve a prolonged abnormally low body fat percentage by DXA or anthropometry, substantial weight loss (5–10% in one month), an attenuation of expected growth and development, an abnormal menstrual cycle in females (amenorrhea > 6 months and/or menarche > 16 years), abnormal hormonal profiles in men, a reduced bone mineral density, a history of ≥1 stress fractures, physical/psychological complications related to LEA or disordered eating, electrocardiogram abnormalities, laboratory abnormalities, a prolonged relative energy deficiency, disordered eating behavior negatively affecting other team members, and a lack of progress in treatment and/or noncompliance. Low-risk criteria include healthy eating habits with appropriate energy availability, normal hormonal and metabolic function, a healthy bone mineral density as expected for the sport, age, and ethnicity, and a healthy musculoskeletal system [2,18].

The further-developed CAT2 builds on CAT1, comprising a four-level traffic light system, and applies primary and secondary indicators to assess the risk of REDs [13,14]. Since CAT2 was not active at the time of the REDs assessment, some diagnostic criteria and indicators for exact CAT2 calculation were often missing. These include blood parameters (e.g., testosterone levels for males, total cholesterol and low-density lipoprotein cholesterol, triiodothyronine), bone mineral density measurements and information on the deviation from the athlete’s previous growth trajectory. Thus, CAT2 scores are not reported.

### 2.4. Statistics

Data were analyzed with SPSS Statistics for Windows, Version 28.0. For continuous variables, the normal distribution was tested with the Shapiro–Wilk test and equality of variances between groups with Levene’s test. Data are reported as the mean ± standard deviation (M ± SD), median and 95% confidence interval (CI). Frequencies are expressed as percentages (%). Parametric data were analyzed with paired and unpaired *t*-tests and non-parametric data with the Wilcoxon-signed rank test when paired, and with the Chi^2^-test and Mann–Whitney U-test, when unpaired. A *p*-value < 0.05 was considered statistically significant. The comparison between the *ADULT*- and *CHILD*-group regarding the baseline characterization was carried out using either the Mann–Whitney U-test, the Chi^2^-test, or the unpaired *t*-test, while the comparison between two-time courses (t_0_/t_1_) was implemented with the Wilcoxon-signed rank test or the paired *t*-test.

## 3. Results

### 3.1. Baseline Characterization of REDs Individuals Reveal Predominantly Female Adolescents and Young Women

The results for the characterization of the study population are presented in detail in Table 1. Fifty-eight athletes aged between 12.5 and 44.0 years with suspected REDs fulfilled the inclusion criteria and participated in the interdisciplinary REDs outpatient consultation at the Dept. of Sports Medicine between January 2019 and December 2022. The athletes were predominantly girls and young women (97%). About two thirds were minors and one third were over the age of 18. More than half of the athletes who presented themselves at the REDs outpatient clinic were conspicuous in the context of the regularly performed national and state team examinations. Another third presented themselves self-initiated or upon the advice of the family or other medical professionals. Among all the athletes, the athletes were predominantly from endurance sports (35%), e.g., middle- or long-distance running, and aesthetic sports (24%), like (rhythmic) gymnastics. The average training volume was 14.9 ± 11.9 h per week. The mean average body fat was low with 6.1 ± 1.5% as determined by the skinfold thickness (triceps, subscapular, and paraumbilical). The BMI analysis, considering the BMI percentiles for children, showed that 59% of the athletes were underweight and 41% normal weight. Most noticeable was the fact that 91% of the female athletes had a menstrual disorder in form of primary (35%) or secondary (50%) amenorrhea or irregular menstruation (6%).

The Dept. of Sports Medicine recommended a removal from training and competition in 7% of the cases and called for a reduction in sports for 45% of the athletes. According to CAT1, a removal from training and competition would have been recommended in 28% of the athletes, and a reduction in over 70%, showing that CAT1 tended to me more conservative than clinical practice. Due to missing variables, CAT2 could not be assessed, since CAT2 was not active at the time of REDs evaluation.

From the total sample, 38 athletes were children and adolescents (*CHILD*-group), and 20 athletes were adults (*ADULT*-group). Overall, the *ADULT*-group and the *CHILD*-group were both similar in their baseline characteristics (Table 1). However, in the *CHILD*-group, the trend was more members of the national or state teams in comparison to the *ADULT*-group (*p* = 0.005). Additionally, more athletes participated in aesthetic sports in the *CHILD*-group, while athletes from the *ADULT*-group performed more endurance sports (*p* = 0.006).

Other characteristics, such as bone stress injuries, occurred in 19%, and the diagnosis of osteoporosis or osteopenia was found in 9%. Analyzing blood parameters, decreased values of leukocytes (52%), hematocrit (37%), and erythrocytes (25%) compared to sex- and age-specific reference ranges, were observed (Supplementary Material S2; Table S1). Deviations from the reference range were overall equally distributed across the *CHILD*- and *ADULT*-group. Electrolytes and minerals including potassium, sodium, and calcium were in the normal reference ranges in >90% of the athletes. In one third of the athletes, decreased iron parameters were observed, while in 48%, the creatine kinase was increased (Supplementary Material S2; Table S1).

### 3.2. Eating Disorders Are Common in Potential REDs Athletes and Dietary Behavior Is Characterized by Plant-Based and Low-Energy-Dense Food

Of the 58 athletes included in this study, 60% had REDs and 40% had an eating disorder, mainly AN (Supplementary Material S3; Figure S1). It is unclear how many of these athletes first had REDs, which then led to an eating disorder, and how many athletes had an eating disorder already, using sports to support their condition. Data on dietary assessment are presented in Supplementary Material S4 (Figure S2). In summary, the athletes had five meals/day on mean average, the food diversity was medium to high, the diet was predominantly plant-based, and over 60% of the chosen foods were low-energy-dense (≤1.5 kcal/g).

### 3.3. Over Two-Third of the Potential REDs Candidates Committed to Longer-Term Treatment

Out of the 58 athletes with suspected REDs, 16 athletes (28%) visited the Dept. of Sports Medicine once (*STOP*-group), while 42 athletes (72%) had longer-term treatment (defined as at least two visits) (*TREAT*-group). Possible reasons range from further intensive treatments at the Departments of Psychosomatic Medicine or Child and Youth Psychiatry, to no treatment motivation over uncertainties with regard to other therapeutic perspectives, to no treatment required in some cases. Regarding the *TREAT*-group, a distinction was made between athletes who already completed their treatment before the year 2023 (*T*_compl_, N = 27; 64%) and athletes who were continuing their treatment in 2023 (*T*_conti_, N = 15; 36%. The *T*_compl_-group were included in the longitudinal analysis to describe changes between t_0_ and t_1_. The baseline characterization of the three groups (all athletes vs. *STOP* vs. *TREAT*) is presented in Table 2. Except for the observed trend of the *STOP*-group having higher weight and waist circumferences, there were no significant differences between the two subgroups in terms of their baseline characteristics.

### 3.4. Effectiveness of Treatment

Twenty-seven athletes completed their treatment (*T*_compl_) and were compared between the baseline (t_0_) and the end of treatment (t_1_), regardless of the treatment duration (14.6 ± 12.4 months) (Table 3). The majority (71%) of the athletes were part of a national or state team. Body weight-related parameters, body fat, waist circumference, and menstrual disorders significantly improved in the course of the program. The mean average weight gain was 7 ± 6 kg. In line, the BMI z-score increased by 0.8 ± 0.7 points.

The analysis of blood cells was overall similar between t_0_ and t_1_ (Supplementary Material S5; Table S2). Overall, many physiological parameters were investigated according to the individual circumstances and requirements of the athletes and not across all athletes over time, leading to small samples sizes and not allowing for comparison.

The treatment at the Dept. of Sports Medicine was continuous, and peaked at the first two and three months after the initial appointment (33% of participants), after one year (26%) and two years (37%). Interdisciplinary follow-up diagnosis and treatment were common. Visits and/or treatment at the Dept. of Gynecology were documented for 19% of the *T*_compl_-group and took place either during the first two months (7%) or after one or two years (7%) after the initial appointment at the Dept. of Sport Medicine. Visits and/or treatment at the Dept. of Psychosomatic Medicine or Child and Youth Psychiatry were recorded for 30% of the athletes and occurred within the first two months after the initial appointment. The total number of appointments at the Dept. of Sports Medicine ranged from a minimum of 2 to 15 visits, while at the Dept. of Psychosomatic Medicine and the Child and Youth Psychiatry, a maximum of 4 appointments were reported (including long-term inpatient treatments).

## 4. Discussion

In this study, 58 athletes with suspected REDs were investigated in clinical practice approaching an outpatient interdisciplinary and multidisciplinary consultation structure for athletes. The athletes were characterized cross-sectionally and longitudinally where applicable, to prove treatment efficacy.

REDs risk evaluation according to CAT1 was difficult to apply in clinical practice since the criteria were too strict on the one hand, but offered on the other hand a high freedom of decision making by medical doctors according to the authors perspective. For example, in this sample, body fat percentages of all female athletes were too low [17], resulting in the yellow categorization according to CAT1. Already, this aspect forced the deviation from the CAT1 recommendations in clinical practice, if the other health aspects of the athlete were inconspicuous. In CAT2, the body fat percentage aspect is not listed anymore. Instead, CAT2 offers clear criteria and instructions with a follow-along plan and Excel sheet for clinical practice, making the clinical expert ratings predictable and independent of the medical doctor in charge. For this study population, CAT2 could not be calculated due to missing variables, since CAT2 was not active at the time of the REDs assessment.

As expected, the population of athletes with REDs was heterogeneous. In line with the literature, REDs particularly affected female athletes in adolescence and young adulthood, often practicing endurance and aesthetic sports while being underweight and with menstrual disorders. Although, about 40% were at normal weight and 60% underweight. Since the average age of onset of menarche is 12.4 years, it can be concluded that menstrual disorders with a rate of 91% are most likely the consequences of extremely low body fat and low energy availability due to high energy expenditure [22,23,24].

Long-term consequences of REDs were found in almost 30% of the cases when applying bone stress injuries, osteoporosis and osteopenia as criteria. Previous studies exploring health associated with REDs and LEA have reported comparable prevalences of stress fractures (27%) [25]. Female athletes with menstrual disorders are more likely to sustain bone stress injuries than athletes with regular menstruation [11,26]. Exploring hormone function and bone turnover markers could have provided additional quantitative variables to assess the long-term consequences of REDs [27].

Strikingly, we demonstrated, for the first time, that around 40% of the athletes did not have REDs but an eating disorder. The proportion may be higher due to underreporting to avoid sport restrictions since the diagnosed eating disorders by the Departments of Psychosomatic Medicine and Child and Youth Psychiatry were based on self-reports by the athletes [28]. In addition, it is unclear how many of the athletes first had REDs, which subsequently led to an eating disorder and, how many athletes had an eating disorder at the onset and used exercise to support their condition [2,29].

However, the food diversity of athletes with REDs was overall medium to high with the proportion of low-energy-dense foods being high. Meal frequency was inconspicuous with five meals per day in the median. The diet of REDs athletes appears less restrictive when compared to patients with anorexia or bulimia nervosa [30]. Thus, most likely a combination of small portion sizes and a high frequency of the consumption of low-energy-dense foods along with the increased energy expenditure through sports are likely to cause energy deficits in this study population.

The longitudinal analysis is promising. At the baseline, about 40% of the athletes were at normal weight, whereas it was over 70% at the end of the treatment. The mean average body weight gain for the whole group was 7 kg over time and the BMI z-score with practical use in adolescents by adjusting for age and gender was 0.8 points. In line, the waist circumference and body fat percentage increased, respectively. These findings demonstrate that an outpatient interdisciplinary and multidisciplinary approach as taken here is an effective approach to address the individual needs of athletes with REDs.

This study has strengths and limitations. A large strength is that a reasonable sample of athletes with suspected REDs was characterized cross-sectionally and longitudinally up to 36 months with a mean average of 15 months. It is reported how an interdisciplinary outpatient consultation program for athletes with REDs can be structured and proves that this low-barrier approach is effective for athletes. This study points out the problems which occurred with CAT1 criteria in clinical practice. However, the advancements in probable coherence between clinical practice and CAT2 could not be investigated in this study population due to missing values, since CAT2 was not active at the time of the REDs assessment. The data collection was heterogeneous due to the extraction from medical letters. Therefore, the data extraction was performed in the most structured procedure as possible. The study population was mostly female and young, which excludes drawing conclusions for males and older REDs athletes. Based on existing literature, athletes participating in endurance and aesthetic sports are at an increased risk of developing eating disorders [8,9,10,11]. Consequently, our study population, which includes a high proportion of athletes from endurance sports (35%) and aesthetic sports (24%), provides a representative sample of individuals at risk for REDs. However, the findings may be less generalizable to athletes from other sports disciplines affected by REDs.

Despite expert interviews, according to ICD-11, the diagnosis of eating disorders partly relies on self-reported data. Therefore, the proportion of eating disorder diagnoses may be higher due to potential underreporting to avoid sport restrictions and revealing disordered eating habits.

## 5. Conclusions

Taken together, the diagnosis and treatment of athletes with REDs can be carried out in a low-barrier interdisciplinary approach if departments and centers work together in a structured pathway approach. The new CAT2 is a tool with clear instructions for clinical practice allowing for predictable decisions independent of the medical expert in charge. However, regarding the treatment of REDs, no standardized recommendations are available yet. It is clear that it needs to be interdisciplinary, but treatment pathways as presented in this manuscript are not officially available yet. Our data may be used as a basis for future research on this important aspect to make treatment evidence-based and predictable. Currently, the treatment of athletes depends largely on decisions made by individual medical experts. This needs improvement to detect and treat existing REDs at an early stage to prevent long-term health consequences and guarantee athletes’ performance.

## Figures and Tables

**Table 1:** Baseline characterization of REDs individuals (N = 58).

	Total N (%) or M ± SD (Median) (95% CI)	CHILD-Group N (%) or M ± SD (Median) (95% CI)	ADULT-Group N (%) or M ± SD (Median) (95% CI)	Statistics for CHILD Versus ADULT Mann–Whitney U-Test/ χ^2^ (df, N)/Unpaired *t*-Test (df)
**Athletes (N)**	58	38 (65.5%)	20 (34.5%)	
Age, years (58)	18.3 ± 5.5 (16.5)	15.5 ± 1.4 (16.0)	23.7 ± 6.4 (22.0)	***p* < 0.001**, U = 0.000,
	(16.9–19.8)	(15.1–16.0)	(20.6–26.7)	***r = −0.82***
Sex (58)				*p* = 0.638
female	56 (96.6%)	37 (97.4%)	19 (95.0%)	
male	2 (3.4%)	1 (2.6%)	1 (5.0%)	
Contacted by (58)				***p* = 0.020**,
medical professionals	17 (29.3%)	8 (21.1%)	9 (45.0%)	χ^2^ = 7.839 (2, N = 58)
private	9 (15.5%)	4 (10.5%)	5 (25.0%)	
team	32 (55.2%)	26 (68.4%)	6 (30.0%)	
Part of a team (58)				***p* = 0.005**,
yes	32 (55.2%)	26 (68.4%)	6 (30.0%)	χ^2^ = 7.821 (1, N = 58)
no	26 (44.8%)	12 (31.6%)	14 (70.0%)	
Type of sports (58)				***p* = 0.006**,
ball/game	4 (6.9%)	3 (7.9%)	1 (5.0%)	χ^2^ = 23.180 (9, N = 58)
endurance	20 (34.5%)	11 (28.9%)	9 (45.0%)	
strength and power	5 (8.6%)	3 (7.9%)	2 (10.0%)	
martial arts	2 (3.4%)	2 (5.3%)	0	
aesthetic	14 (24.1%)	14 (36.8%)	0	
technical	1 (1.7%)	1 (2.6%)	0	
multiple	9 (15.5%)	3 (7.9%)	6 (30.0%)	
none	3 (5.2%)	1 (2.6%)	2 (10.0%)	
Training volume, h/week (54)	14.9 ± 11.9 (10.3)	18.0 ± 13.4 (13.0)	9.7 ± 6.4 (7.8)	*p* = 0.060
	(11.6–18.1)	(13.3–22.6)	(6.7–12.2)	
Weight, kg (56)	49.0 ± 7.9 (48.3)	47.7 ± 7.3 (46.5)	51.6 ± 8.5 (49.9)	*p* = 0.078
	(46.9–51.1)	(45.2–50.1)	(47.5–55.6)	
Height, m (56)	166.2 ± 7.8 (165.0)	166.0 ± 7.4 (166.5)	166.5 ± 8.7 (163.4)	*p* = 0.840
	(164.1–168.3)	(163.6–168.5)	(162.3–170.7)	
BMI, kg/m^2^ (56)	17.7 ± 2.2 (17.3)	17.2 ± 1.8 (16.6)	18.6 ± 2.8 (18.0)	*p* = 0.061
	(17.1–18.3)	(16.6–17.8)	(17.2–19.9)	
BMI classification (56)				*p* = 0.910
underweight	33 (58.9%)	22 (59.5%)	11 (57.9%)	
normal weight	23 (41.1%)	15 (40.5%)	8 (42.1%)	
BMI z-Score (44)	−1.7 ± 1.2 (−1.7)	−1.5 ± 1.0 (−1.6)	−2.4 ± 0.2.0 (−1.9)	*p* = 0.163
	(−2.0–−1.3)	(−1.9–−1.2)	(−4.2–−0.5)	
Skinfold thickness, mm (55)				
triceps	6.0 ± 3.0 (5.0)	5.8 ± 2.3 (5.0)	6.6 ± 4.1 (6.5)	*p* = 0.709
	(5.2–6.8)	(5.0–6.5)	(4.5–8.6)	
subscapular	7.0 ± 2.6 (6.0)	6.8 ± 2.4 (6.0)	7.3 ± 2.9 (8.0)	*p* = 0.636
	(6.3–7.7)	(6.0–7.6)	(5.8–8.7)	
paraumbilical	8.3 ± 4.4 (7.0)	8.2 ± 4.6 (6.0)	8.6 ± 4.1 (8.5)	*p* = 0.569
	(7.2–9.5)	(6.7–9.7)	(6.6–10.6)	
Body fat, % (55)	6.1 ± 1.5 (5.7)	6.0 ± 1.3 (5.7)	6.3 ± 1.8 (6.5)	*p* = 0.629
	(5.7–6.5)	(5.6–6.5)	(5.4–7.2)	
Waist circumference, cm (55)	67.2 ± 5.5 (68.0)	66.2 ± 4.9 (66.5)	69.2 ± 6.1 (69.5)	*p* = 0.054
	(65.7–68.6)	(64.5–67.8)	(66.1–72.2)	
Menstrual disorders (54)				*p* = 0.678
none	5 (9.2%)	3 (8.1%)	2 (11.8%)	
primary amenorrhea	19 (35.2%)	15 (40.5%)	4 (23.5%)	
secondary amenorrhea	27 (50.0%)	17 (46.0%)	10 (58.8%)	
irregular menstruation	3 (5.6%)	2 (5.4%)	1 (5.9%)	
**Multidisciplinary recommendations**				
Training recommendation (58)				*p* = 0.781
prohibition	4 (6.9%)	2 (5.3%)	2 (10.0%)	
restriction	26 (44.8%)	17 (44.7%)	9 (45.0%)	
permission	28 (48.3%)	19 (50.0%)	9 (45.0%)	
Gynecology (56)				*p* = 0.558
no	23 (41.1%)	14 (37.8%)	9 (47.4%)	
yes	18 (32.1%)	11 (29.7%)	7 (36.8%)	
planned	12 (21.4%)	10 (27.0%)	2 (10.5%)	
already in treatment	3 (5.4%)	2 (5.4%)	1 (5.3%)	
Psychosomatic Medicine/Child and Youth Psychiatry (58)				
no	34 (58.6%)	24 (63.2%)	10 (50.0%)	*p* = 0.071
yes	15 (25.9%)	6 (15.8%)	9 (45.0%)	
planned	2 (3.4%)	2 (5.3%)	0	
already in treatment	7 (12.1%)	6 (15.8%)	1 (5.0%)	

Notes: The BMI z-score only considered children and adolescents as well as adults up to the age of 20. Adults > 20 years were excluded from the analysis (N = 12). In the analysis of menstrual disorders, all male athletes (N = 2) and female athletes with use of contraceptive pills (N = 1) were excluded; in the analysis of recommendations for gynecology, all male athletes (N = 2) were excluded. Abbreviations: BMI: body mass index. Statistics: CI: confidence interval, df: degrees of freedom, M: mean, U: Mann–Whitney U-test, χ^2^: χ^2^ test, *r*: effect size (for Mann–Whitney U-test), and SD: standard deviation. *p* < 0.05 is considered statistically significant and are marked boldly.

**Table 2:** Baseline characterization of all REDs individuals (N = 58) vs. *STOP*-group (N = 16) vs. TREAT-group (N = 42).

	Total N (%) or M ± SD (Median) (95% CI)	STOP-Group N (%) or M ± SD (Median) (95% CI)	TREAT-Group N (%) or M ± SD (Median) (95% CI)	Statistics for STOP Versus TREAT Mann–Whitney U-Test/ χ^2^ (df, N)/Unpaired *t*-Test (df)
**Athletes (N)**	58	16 (27.6%)	42 (72.4%)	
Age, years (58)	18.3 ± 5.5 (16.5)	20.2 ± 6.2 (17.5)	17.6 ± 5.1 (22.0)	*p = 0.150*
	(16.9–19.8)	(16.9–23.5)	(16.0–19.2)	
Age group (58)				*p = 0.125*
<18 years	38 (65.5%)	8 (50.0%)	30 (71.4%)	
≥18 years	20 (34.5%)	8 (50.0%)	12 (28.6%)	
Contacted by (58)				*p = 0.555*
medical professionals	17 (29.3%)	6 (37.5%)	11 (26.2%)	
private	9 (15.5%)	3 (18.8%)	6 (14.3%)	
team	32 (55.2%)	7 (43.8%)	25 (59.5%)	
Part of a team (58)				*p = 0.280*
yes	32 (55.2%)	7 (43.8%)	25 (59.5%)	
no	26 (44.8%)	9 (56.3%)	17 (40.5%)	
Type of sports (58)				*p = 0.193*
ball/game	4 (6.9%)	2 (12.5%)	2 (4.8%)	
endurance	20 (34.5%)	3 (18.8%)	17 (40.5%)	
strength and power	5 (8.6%)	3 (18.8%)	2 (4.8%)	
martial arts	2 (3.4%)	0	2 (4.8%)	
aesthetic	14 (24.1%)	3 (18.8%)	11 (26.2%)	
technical	1 (1.7%)	0	1 (2.4%)	
multiple	9 (15.5%)	4 (25.0%)	5 (11.9%)	
none	3 (5.2%)	1 (6.3%)	2 (4.8%)	
Training volume, h/week (54)	14.9 ± 11.9 (10.3)	15.5 ± 11.3 (13.3)	14.6 ± 12.3 (9.1)	*p* = 0.576
	(11.6–18.1)	(9.5–21.6)	(10.6–18.7)	
Weight, kg (56)	49.0 ± 7.9 (48.3)	53.2 ± 8.7 (51.5)	47.3 ± 6.9 (46.4)	***p* = 0.010**, t(54) = 2.665
	(46.9–51.1)	(48.5–57.8)	(45.1–49.5)	
Height, m (56)	166.2 ± 7.8 (165.0)	169.3 ± 8.0 (168.8)	165.0 ± 7.5 (163.0)	*p* = 0.062
	(164.1–168.3)	(165.0–173.5)	(162.6–167.3)	
BMI, kg/m^2^ (56)	17.7 ± 2.2 (17.3)	18.5 ± 2.4 (18.0)	17.4 ± 2.1 (16.6)	*p* = 0.052
	(17.1–18.3)	(17.2–19.8)	(16.7–18.0)	
BMI classification (56)				*p* = 0.390
underweight	33 (58.9%)	8 (50.0%)	25 (62.5%)	
normal weight	23 (41.1%)	8 (50.0%)	15 (37.5%)	
BMI z-Score (44)	−1.7 ± 1.2 (−1.7)	−1.2 ± 0.9 (−1.3)	−1.8 ± 1.2 (−1.8)	*p = 0.194*
	(−2.0–−1.3)	(−1.9–−0.6)	(−2.2–−1.4)	
Skinfold thickness, mm (55)				
triceps	6.0 ± 3.0 (5.0)	7.1 ± 3.6 (6.0)	5.6 ± 2.7 (5.0)	*p* = 0.106
	(5.2–6.8)	(5.2–9.0)	(4.7–6.4)	
subscapular	7.0 ± 2.6 (6.0)	7.3 ± 2.6 (6.5)	6.8 ± 2.6 (6.0)	*p* = 0.517
	(6.3–7.7)	(5.9–8.6)	(6.0–7.7)	
paraumbilical	8.3 ± 4.4 (7.0)	9.0 ± 3.9 (8.0)	8.1 ± 4.6 (6.0)	*p* = 0.271
	(7.2–9.5)	(6.9–11.0)	(6.6–9.6)	
Body fat, % (55)	6.1 ± 1.5 (5.7)	6.5 ± 1.5 (6.3)	5.9 ± 1.5 (5.7)	*p = 0.225*
	(5.7–6.5)	(5.7–7.2)	(5.5–6.4)	
Waist circumference, cm (55)	67.2 ± 5.5 (68.0)	69.5 ± 5.4 (69.0)	66.2 ± 5.3 (65.0)	***p* = 0.039**, t(53) = 2.120
	(65.7–68.6)	(66.7–72.4)	(64.5–67.9)	
Menstrual disorders (54)				*p* = 0.660
none	5 (9.2%)	2 (15.4%)	3 (7.3%)	
primary amenorrhea	19 (35.2%)	3 (23.1%)	16 (39.0%)	
secondary amenorrhea	27 (50.0%)	7 (53.8%)	20 (48.8%)	
irregular menstruation	3 (5.6%)	1 (7.7%)	2 (4.9%)	
**Multidisciplinary recommendations**				
Training recommendation (58)				*p* = 0.434
prohibition	4 (6.9%)	0	4 (9.5%)	
restriction	26 (44.8%)	8 (50.0%)	18 (42.9%)	
permission	28 (48.3%)	8 (50.0%)	20 (47.6%)	
Gynecology (56)				*p* = 0.331
no	23 (41.1%)	9 (60.0%)	14 (34.1%)	
yes	18 (32.1%)	3 (20.0%)	15 (36.6%)	
planned	12 (21.4%)	2 (13.3%)	10 (23.8%)	
already in treatment	3 (5.4%)	1 (6.7%)	2 (4.9%)	
Psychosomatic Medicine/Child and Youth Psychiatry (58)				*p* = 0.103
no	34 (58.6%)	9 (56.3%)	25 (59.5%)	
yes	15 (25.9%)	7 (43.7%)	8 (19.0%)	
planned	2 (3.4%)	0	2 (4.8%)	
already in treatment	7 (12.1%)	0	7 (16.7%)	

Notes: The BMI z-score only considered children and adolescents as well as adults up to the age of 20. Adults > 20 years were excluded from the analysis (N = 12). In the analysis of menstrual disorders, all male athletes (N = 2) and female athletes with use of contraceptive pills (N = 1) were excluded; in the analysis of recommendations for gynecology, all male athletes (N = 2) were excluded. Abbreviations: BMI: body mass index. Statistics: CI: confidence interval, df: degrees of freedom, M: mean, U: Mann–Whitney U-test, χ^2^: χ^2^ test, *r*: effect size (for Mann–Whitney U-test), and SD: standard deviation. *p* < 0.05 is considered statistically significant and are marked boldly.

**Table 3:** Comparison of characteristics of *T*_compl_ (N = 27) at t_0_ and t_1_.

	t_0_ N (%) or M ± SD (Median) (95% CI)	t_1_ N (%) or M ± SD (Median) (95% CI)	Statistics for t_0_ Versus t_1_ Wilcoxon, Paired *t*-Test (df)
**Athletes (N)**	27	27	
Age, years (27)	16.3 ± 2.7 (16.0)	17.6 ± 2.8 (17.5)	***p* < 0.001**, *r* = −0.80
	(15.3–17.4)	(16.5–18.7)	(N = 27)
Sex (27)			
female	26 (96.3%)		
male	1 (3.7%)		
Treatment duration, months (27)	14.6 ± 12.4 (15.0)		
	(9.7–19.5)		
Weight, kg (21)	47.3 ± 7.4 (46.5)	54.4 ± 7.6 (53.2)	***p* < 0.001**, t(20) = −5.370
	(43.9–50.7)	(50.9–57.8)	
Height, m (21)	164.9 ± 7.3 (164.7)	167.0 ± 6.2 (166.0)	***p* = 0.014**, t(20) = −2.680
	(161.6–168.3)	(164.2–169.8)	
BMI, kg/m^2^ (21)	17.3 ± 2.0 (16.6)	19.5 ± 2.3 (19.2)	***p* < 0.001**, t(20) = −5.807
	(16.4–18.2)	(18.4–20.5)	
BMI classification (21)			***p* = 0.014**, *r* = −0.53
underweight	12 (57.1%)	6 (28.6%)	(N = 21)
normal weight	9 (42.9%)	15 (71.4%)	
BMI z-score (19)	−1.4 ± 1.0 (1.4)	−0.6 ± 0.8 (−0.7)	***p* < 0.001**, t(18) = −5.182
	(−1.9–−0.9)	(−1.0–−0.2)	
Weight change, kg (21)	7.1 ± 6.0 (6.5)		
	(4.3–9.8)		
Weight change (21)			
increase	20 (95.2%)		
decrease	1 (4.8%)		
BMI z-score change (19)	0.8 ± 0.7 (0.6)		
	(0.5–1.2)		
Skinfold thickness, mm (18)			
triceps	5.9 ± 3.1 (5.5)	6.5 ± 3.0 (6.0)	*p* = 0.423
	(4.4–7.5)	(5.0–8.0)	
subscapular	7.1 ± 2.6 (6.0)	7.9 ± 2.2 (7.5)	*p* = 0.124
	(5.8–8.4)	(6.9–9.0)	
paraumbilical	9.2 ± 5.0 (8.0)	10.8 ± 3.9 (10.5)	*p* = 0.151
	(6.8–11.7)	(8.9–12.8)	
Body fat, % (18)	6.2 ± 1.4 (5.9)	6.9 ± 1.1 (6.9)	***p* = 0.021**, t(17) = −2.547
	(5.5–7.0)	(6.4–7.5)	
Body fat change, % (18)	0.7 ± 1.1 (0.7)		
	(0.1–1.2)		
Body fat change (18)			
increase	12 (66.7%)		
decrease	5 (27.8%)		
unchanged	1 (5.6%)		
Waist circumference, cm (18)	66.3 ± 5.1 (65.3)	68.9 ± 4.0 (68.5)	***p* = 0.023**, t(17) = −2.495
	(63.8–68.9)	(66.9–70.9)	
Menstrual disorders (19)			***p* = 0.047**, *r* = −0.46
none	1 (5.3%)	9 (47.4%)	(N = 19)
primary amenorrhea	8 (42.1%)	5 (26.3%)	
secondary amenorrhea	9 (47.3%)	3 (15.8%)	
irregular menstruation	1 (5.3%)	2 (10.5%)	
**Multidisciplinary follow-up**			
Recommendation gynecology (26)			
No	11 (42.3%)		
Yes	6 (23.1%)		
Planned	7 (26.9%)		
Already in treatment	2 (7.7%)		
Presentation gynecology (26)			
No	17 (65.4%)		
Yes	5 (19.2%)		
Follow-up (2023)	4 (15.4%)		
Recommendation Psychosomatic Medicine, Child and Youth Psychiatry (27)			
No	17 (63.0%)		
Yes	4 (14.8%)		
Planned	2 (7.4%)		
Already in treatment	4 (14.8%)		
Presentation Psychosomatic Medicine, Child and Youth Psychiatry (27)			
No	19 (70.4%)		
Yes	8 (29.6%)		

Notes: The *T*_compl_-group (N = 27) included all the athletes who completed their treatment before the year 2023. The BMI z-score only considered children and adolescents as well as adults up to the age of 20. Adults > 20 years were excluded from the analysis (N = 2). In the analysis of menstrual disorders, the male athlete (N = 1) was excluded. Abbreviations: BMI: body mass index, t_0_: before treatment, t_1_: after treatment. Statistics: CI: confidence interval, df: degrees of freedom, M: mean, *r*: effect size (for Wilcoxon test), SD: standard deviation. *p* < 0.05 is considered statistically significant and are marked boldly.

## Data Availability

Authors can be contacted directly for questions regarding the data set.

## Supplementary Materials

The following supporting information can be downloaded at https://www.mdpi.com/article/10.3390/nu17020228/s1: Table S1: Baseline blood parameters of REDs individuals and assessment according to gender- and age-specific reference ranges; Figure S1: Percentage distribution of diagnosis regarding REDs and eating disorders at t_0_; Figure S2: Percentage distribution of food groups ingested over three days based on frequencies; and Table S2: Comparison of blood parameters in *T*_compl_-group between t_0_ and t_1_.
