# Supplementary material for: Relative Energy Deficiency in Sport—Multidisciplinary Treatment in Clinical Practice
Source: Nutrients. 2025 Jan 9;17(2):228. doi: 10.3390/nu17020228 (PMC11767807; doi:10.3390/nu17020228)
Supplement: Supplementary file 1 [file nutrients-17-00228-s001.zip › nutrients-3399655-supplementary.pdf]

Supplementary Materials

Relative Energy Deficiency in Sport (REDs) – Multidisciplinary Treatment in Clinical Practice

Andrea Meyer [1,2], Daniel Haigis [2], Bea Klos [1], Stephan Zipfel [1], Gaby Resmark [1], Katharina Rall [3], Katharina Dreser [3], Daniela Hagmann [4,5], Andreas Niess [2], Christine Kopp [2,*] and Isabelle Mack [1]

[1] Department of Psychosomatic Medicine and Psychotherapy, University Hospital Tübingen, Tübingen, Germany
[2] Department of Sports Medicine, University Hospital Tübingen, Tübingen, Germany
[3] Department of Womens' Health, University of Tübingen, Tübingen, Germany
[4] Department of Child and Adolescent Psychiatry, Psychosomatics and Psychotherapy, University Hospital Tübingen, Tübingen, Germany
[5] German Center for Mental Health, Tübingen, Germany
* Correspondence: Christine.Kopp@med.uni-tuebingen.de

Supplementary Material S1. Methods

Blood Parameters

Partial blood examinations were carried out. The venous blood collection of the athletes was conducted following clinical standard procedure, adhering to guidelines specific to the parameter under investigation [22]. The following blood parameters were mainly examined in laboratory tests: small blood count (erythrocytes, hemoglobin concentrations, hematocrit, leukocytes, platelets) and substrates (thyroid stimulating hormone, glucose, potassium, sodium, calcium, iron, ferritin, 25-OH-vitamin D and creatine kinase).

Dietary Protocols

Dietary protocols were analyzed if available. These protocols were heterogeneous in terms of reporting and details on food quantities. Therefore, data were assessed qualitatively based on the frequency of meal and snacks, frequency of twelve food groups to calculate the dietary diversity over three days, as well as the evaluation of food items on basis of energy density (kilocalories per gram).

In detail, a randomization of the week and weekend days was implemented. A total of three days was evaluated, including two days during the week and one weekend day. The following information was extracted from the dietary protocols: frequency of meal and snacks, individual components of the dish or as a complete meal and the associated energy density of the individual foods based on the Federal Food Code. Meal frequency was assessed by counting all meals throughout the day. As an indicator of nutrient adequacy, a dietary diversity score was evaluated by counting the frequencies of consumed food groups over three days [23]. In total twelve food groups that align with the food groups in the European Prospective Investigation of Cancer (EPIC) Food Frequency Questionnaire (FFQ) were considered [24]. The following food groups were included: 1) meat and meat products, 2) fish and fish products, 3) milk and dairy products, 4) eggs and egg dishes, 5) cereal and cereal products, 6) potatoes, 7) fats and oils, 8) nuts and seeds, 9) soups and sauces, 10) sugar, preserves and snacks, 11) vegetables and 12) fruits. Beverages were excluded because the athletes mostly only drank water, or the beverages were not considered in the dietary protocols. Reported fruit juice (e.g. orange juice), fruit smoothies and shakes (e.g. banana shake) were included within the fruit group. When a complete meal was given (e.g. spaghetti bolognese), the individual food components were considered as far as possible using standard recipes. Afterwards, the components of the meal were assigned to the associated food group. Dietary diversity was assessed by the number of food groups each individual consumed over three days. Dietary diversity was classified as low (1-4 food groups), medium (5-8 food groups) and high (9-12 food groups) [23]. Finally, the foods were categorized according to their energy density. The dietary energy

density of food is defined as the energy content (in kcal or kJ) per unit of weight (g or 100g) [25]. Energy density was classified as follows: foods with an energy density up to 1.5 kcal/g have a low energy density and are labeled green. Foods with a medium energy density between 1.5 and 2.5 kcal/g are labeled yellow, and foods with a high energy density above 2.5 kcal/g are classified as red [26].

Supplementary Material S2. Results

Table S1: Baseline blood parameters of REDs individuals and assessment according to gender- and age-specific reference ranges.

	**Total**<br>M±SD (Median)<br>(95% CI)	***CHILD*-group**<br>M±SD (Median)<br>(95% CI)	***ADULT*-group**<br>M±SD (Median)<br>(95% CI)	**Comparison to reference range**<br>N
**Blood parameters (N)**				
Erythrocytes, Mio/µl (52)	4.3±.4 (4.4)<br>(4.2-4.4)	4.4±.3 (4.4)<br>(4.3-4.5)	4.2±.4 (4.3)<br>(3.9-4.4)	↓ N=13<br>↔ N=39
Haemoglobin, g/dl (52)	12.9±1.2 (13.1)<br>(12.5-13.2)	13.0±1.3 (13.2)<br>(12.6-13.4)	12.6±1.2 (13.0)<br>(12.0-13.3)	↓ N=11<br>↔ N=41
Haematocrit, % (52)	37.5±3.4 (37.9)<br>(36.5-38.4)	37.8±3.4 (38.4)<br>(36.7-39.0)	36.7±3.4 (37.7)<br>(34.8-38.5)	↓ N=19<br>↔ N=33
Leucocytes, 1/µl (52)	4844.8±1312.5 (4650.0)<br>(4479.4-5210.2)	4960.6±1134.9 (4725.0)<br>(4576.5-5344.6)	4584.4±1657.3 (4365.0)<br>(3701.2-5467.5)	↓ N=27<br>↔ N=25
Platelets, 1000/µl (52)	253.3±52.8 (246.5)<br>(238.6-267.0)	251.1±46.2 (246.5)<br>(235.4-266.7)	258.3±66.8 (246.5)<br>(222.7-293.8)	↔ N=52
TSH, mU/l (39)	1.9±.9 (1.8)<br>(1.6-2.2)	2.0±1.0 (1.9)<br>(1.6-2.4)	1.7±.7 (1.8)<br>(1.3-2.2)	↑ N=1<br>↔ N=38
Glucose, mg/dl (42)	80.5±17.5 (79.5)<br>(75.0-85.9)	77.8±7.7 (77.0)<br>(74.8-80.7)	86.5±29.2 (85.0)<br>(68.8-104.1)	↓ N=7<br>↑ N=2<br>↔ N=33
Potassium, mmol/l (50)	3.9±.3 (3.9)<br>(3.8-4.0)	4.0±.3 (3.9)<br>(3.8-4.1)	3.9±.3 (3.9)<br>(3.7-4.0)	↓ N=2<br>↑ N=1<br>↔ N=47
Sodium, mmol/l (50)	139.8±1.9 (140.0)<br>(139.3-140.3)	140.1±1.7 (140.0)<br>(139.5-140.7)	139.1±2.1 (140.0)<br>(138.0-140.2)	↓ N=1<br>↔ N=49
Calcium, mmol/l (36)	2.3±.1 (2.3)<br>(2.3-2.4)	2.4±.1 (2.3)<br>(2.3-2.4)	2.3±.1 (2.3)<br>(2.2-2.4)	↔ N=36
Iron, µg/dl (48)	81.1±32.1 (77.0)<br>(71.7-90.4)	80.9±32.0 (77.0)<br>(69.9-91.8)	81.6±33.8 (73.0)<br>(61.2-102.0)	↓ N=13<br>↑ N=2<br>↔ N=33
Ferritin, µg/dl (50)	3.7±3.0 (2.7)<br>(2.9-4.6)	3.9±3.2 (2.9)<br>(2.8-5.0)	3.3±2.7 (2.5)<br>(1.7-4.9)	↓ N=5<br>↑ N=2<br>↔ N=43
25-OH-Vit. D, nmol/l (19)	71.4±25.5 (67.9)<br>(59.1-83.7)	66.1±24.8 (65.0)<br>(52.3-79.8)	91.5±18.5 (92.5)<br>(62.0-120.9)	↓ N=4<br>↔ N=15
CK, U/l (44)	254.2±309.1 (172.0)<br>(160.2-348.2)	267.6±346.4 (170.0)<br>(140.5-394.6)	222.4±202.2 (174.0)<br>(100.2-344.5)	↑ N=21<br>↔ N=23

Notes: In the analysis of blood parameters of the CHILD-group (<18 years; N=38) and ADULT-group (≥18 years; N=20), gender- and age-specific reference ranges were considered. ↓ represented a decreased parameter compared to the reference range, ↑ represented an increased parameter compared to the reference range and ↔ represented parameters within normal range (reference range).
Abbreviations: CK: Creatine Kinase, TSH: Thyroid Stimulating Hormone, Vit.: Vitamin. Statistics: CI: Confidence Interval, M: Mean, Min: Minimum, Max: Maximum, SD: Standard Deviation.

Supplementary Material S3. Results

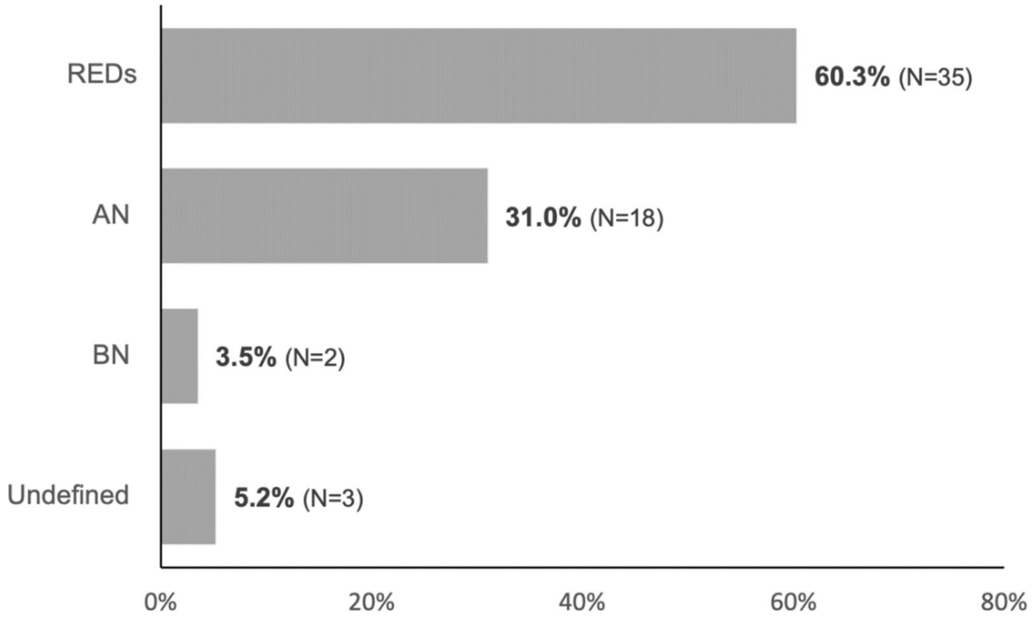

Figure S1: Percentage distribution of diagnosis regarding REDs and eating disorders at t0. Percentage of Relative Energy Deficiency in Sport (REDs) affected athletes, athletes with Anorexia Nervosa (AN), Bulimia Nervosa (BN) or with undefined eating disorders. Undefined eating disorders include, e.g. no specified eating disorders.

Supplementary Material S4. Results

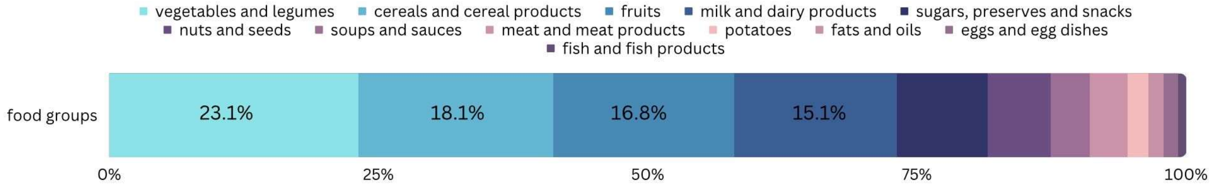

Figure S2: Percentage distribution of food groups ingested over three days based on frequencies. Percentage of twelve food groups consumed most frequently over three days (vegetables and legumes, cereals and cereal products, fruits and milk and dairy products). Percentage of the other eight food groups were not included for clarity.

Supplementary Material S5. Results

Table S1: Comparison of blood parameters in T_{compl}-group between t_0 and t_1.

	**t_0** N (%) or M±SD (Median) (95% CI)	**t_1** N (%) or M±SD (Median) (95% CI)	**Statistics for t_0 versus t_1** Wilcoxon test, paired t-test (df)
**Blood parameters (N)**			
Erythrocytes, Mio/µl (15)	4.5±.4 (4.5) (4.3–4.7)	4.6±.3 (4.6) (4.4-4.7)	*p*=.334
Haemoglobin, g/dl (15)	13.1±1.6 (13.3) (12.2-14.0)	13.3±1.2 (13.5) (12.7-14.0)	*p*=.669
Haematocrit, % (15)	38.2±4.0 (38.7) (35.9-40.4)	38.7±2.9 (38.3) (37.1-40.4)	*p*=.670
Leukocytes, 1/µl (15)	5254.0±1262.3 (4750.0) (4555.0-5953.0)	5390.7±1323.1 (5100.0) (4657.9-6123.4)	*p*=.647
Platelets, 1000/µl (15)	266.1±36.2 (257.0) (246.0-286.1)	280.9±53.2 (290.0) (251.4-310.3)	*p*=.174
TSH, mU/l (7)	2.7±1.3 (2.1) (1.5-4.0)	1.7±.9 (1.4) (.8-2.6)	*p*=.126
Glucose, mg/dl (11)	75.8±7.2 (77.0) (71.0-80.6)	85.9±8.5 (86.0) (80.2-91.6)	***p*=.009,** t(10)=-3.259
Potassium, mmol/l (16)	3.9±.3 (3.8) (3.8-4.1)	3.9±.3 (3.9) (3.7-4.1)	*p*=.875
Sodium, mmol/l (16)	140.4±1.7 (140.5) (139.5-141.3)	139.2±1.5 (139.0) (138.4-140.0)	***p*=.016**, t(15)=2.700
Iron, µg/dl (15)	92.5±40.8 (95.0) (69.9-115.1)	72.1±35.1 (75.0) (52.7-91.6)	*p*=.189
Ferritin, µg/dl (15)	3.4±3.1 (2.5) (1.7-5.1)	1.9±.9 (1.6) (1.4-2.4)	***p*=.023**, r=-.59 (N=15)
Creatine Kinase, U/l (15)	409.1±477.5 (247.0) (144.6-673.5)	227.9±138.1 (210.0) (151.4-304.3)	*p*=.211

Notes: Athletes included in the T_{compl}-group (N=27) had completed their treatment before year 2023. Due to missing data for t_1, no comparison could be performed between t_0 and t_1 for 25-OH-Vitamin D and Calcium. Significant results are marked boldly.

Abbreviations: TSH: Thyroid Stimulating Hormone, t_0: before treatment, t_1: after treatment. Statistics: CI: Confidence Interval, df: degrees of freedom, M: Mean, r: effect size (for Wilcoxon-test), SD: Standard Deviation. $p<.05$ is considered statistically significant.
